# Effect of high-intensity interval training on metabolic parameters in women with polycystic ovary syndrome: A systematic review and meta-analysis of randomized controlled trials

**DOI:** 10.1371/journal.pone.0245023

**Published:** 2021-01-19

**Authors:** Isis Kelly dos Santos, Fernando Antônio Santana de Souza Nunes, Victor Sabino Queiros, Ricardo Ney Cobucci, Pedro Bruch Dantas, Gustavo Mafaldo Soares, Breno Guilherme de Araújo Tinoco Cabral, Tecia Maria de Oliveira Maranhão, Paulo Moreira Silva Dantas

**Affiliations:** 1 Graduate Program in Health Sciences - Federal University of Rio Grande do Norte, Natal, Brazil; 2 Graduate Program in Physical Education - Federal University of Rio Grande do Norte, Natal, Brazil; 3 Biotechnology Graduate Program and Medical School - Potiguar University, Natal, Brazil; 4 Graduate in Biomedicine - Federal University of Rio Grande do Norte, Natal, Brazil; 5 Department of Toco-Gynecology - Federal University of Rio Grande do Norte, Natal, Brazil; 6 Department of Physical Education - Federal University of Rio Grande do Norte, Natal, Brazil; Zhejiang University College of Life Sciences, CHINA

## Abstract

**Background:**

Our aim was to assess the effect of high-intensity interval training (HIIT) on metabolic parameters and body composition in women with polycystic ovary syndrome (PCOS).

**Methods and analysis:**

A systematic review and meta-analysis of randomized controlled trials was conducted using Embase, MEDLINE (via Ovid), PubMed, Sport Discus, Scopus, Web of Science, Cochrane Library and Google Scholar (advanced feature) up to September 2020. Two authors independently screened citations and determined the risk of bias and quality of evidence using the Grading of Recommendations Assessment, Development and Evaluation (GRADE). Meta-analyses were conducted using random effects model.

**Results:**

Seven trials (n = 423) were included in the systematic review. The studies included HIIT interventions vs. moderate exercise or control groups. Most studies were small (average 32, range 24–110 participants) and of relatively short duration (10–16 weeks). The training intensity was performed between 90% and 95% of the maximum heart rate, three times a week, for at least 10 weeks. Insulin resistance, measured using homeostatic model assessment for insulin resistance (HOMA-IR), and body mass index (BMI) showed a significant decrease (MD −0.57; 95% CI, −0.98 to −0.16, p = 0.01), (MD −1.90, 95% CI −3.37, −0.42, p = 0.01) with moderate and high certainty of evidence, respectively.

**Conclusion:**

Results support that HIIT alone is effective for reducing HOMA-IR and BMI in women with PCOS. However, evidence is limited to discern the effect of HIIT on other outcomes. Future studies with a longer duration (> 16 weeks), larger sample sizes and other outcomes are needed.

## 1. Background

Polycystic ovary syndrome (PCOS) is a common disorder with clinical features, such as irregular menstrual cycles, anovulation oligo (reduced ovulation), biochemical hyperandrogenism (elevated hormones or androgens), clinical hyperandrogenism (hirsutism), and infertility, that affects women of reproductive age [1,2]. Women with PCOS may experience an increased risk of developing cardiovascular disease, type 2 diabetes mellitus, obesity, and metabolic syndrome (grouping of risk factors), in addition to depression and anxiety [3,4].

Although the etiology of PCOS is unknown, there are several first-line treatments that assist in the clinical factors of PCOS as ovulation inducing agents. The preventive and therapeutic use of insulin sensitizing agents is increasingly being adopted, alone or in combination with other pharmacological options [5,6]. Lifestyle intervention (including physical activity/exercise and diet) is an effective and low-cost form of non-pharmacological treatment that improves cardiovascular risk factors and helps with weight loss and hormonal dysfunction [7].

Multiples systematic reviews and meta-analyses provide summaries of the beneficial effects of exercise on metabolic, cardiovascular, and psychological parameters in women with PCOS. When observing studies on high-intensity interval training (HIIT) involving high intensity intervals (i.e., ≥ 90% of HRmax) separated by low intensity active rest periods (i.e., ≤ 75% of HRmax), it was found that it helped in improving insulin sensitivity and cardiorespiratory fitness in clinical populations, including women with PCOS [8–10]. Despite the studies showing significant results after interventions with HIIT, there are still some gaps regarding the effect of this intervention on clinical markers in women with PCOS [2,11–13].

To our knowledge, no systematic reviews have investigated the effects of HIIT on clinical markers in women with PCOS. Therefore, this systematic review aimed to synthesize available evidence on the effects of HIIT on metabolic parameters and body composition in women with PCOS.

## 2. Methods

### Protocol registration

This is a systematic review and meta-analysis of randomized clinical trials on the effect of HIIT interventions on metabolic parameters and body composition of women with PCOS. We followed guidelines provided under PRISMA (Preferred Reporting Items for Systematic Reviews and Meta-Analyses) for conducting and reporting systematic reviews [7]. We registered the review on PROSPERO (2020 CRD42020173105) and provided updates to the protocol, when appropriate.

### Eligibility criteria (concepts)

We identified peer-reviewed publications that included the following criteria. **Population**: women (18–40 years) diagnosed with PCOS based on the Rotterdam criteria, National Institutes of Health criteria (NIH); **Intervention**: We only included randomized controlled trials (RCTs) that tested HIIT [*a popular fitness program that encompasses short bouts of high intensity exercise (80–100% of peak heart rate) interspersed with active recovery* [14]], which was at least 4 weeks in duration; **Comparator**: We did not restrict inclusion by type of comparator; however, the trial needed to include an HIIT group only arm; **Outcome**: metabolic parameters (primary) and body composition (secondary). We excluded trials with adolescents (mean age <18 years) and animal studies. The last search was conducted in September 2020.

### Information sources and searches

We included all peer-reviewed publications of RCTs, regardless of language or year of publication. We searched the following databases: Embase, MEDLINE (*via* Ovid), PubMed, Sport Discus, Scopus, Web of Science, Cochrane Library, and Google Scholar (advanced feature). We developed search strategies using a combination of Medical Subject Headings (MeSH) terms and text words related to HIIT or exercise interventions for women with PCOS. Table 1 provides an example of our search strategy.

**Table 1:** MEDLINE (Ovid) search strategy.

Search strategy
1. polycystic ovary syndrome
2. PCOS
3. exercise.mp
4. high intensity training
5. high intensity exercise*
6. high intensity activit*
7. high intensity intermittent training
8. intensity intermittent exercise*
9. high intensity intermittent activit*
10. high intensity interval training
11. high intensity interval exercise*
12. high intensity interval activit*
13. randomized controlled trial
14. RCT
15. #1 OR #2
16. #3 OR #4 OR #5 OR #6 OR #7 OR #8 OR #9 OR #10 OR #11 OR #12
17. #13 OR #14
18. #15 AND #16 AND #17

### Study selection (screening, Level 1, Level 2)

We used Ryyan QCRI (Ryyan QCRI, Qatar Computing Research Institute, HBKU, Doha, Qatar) [15] for screening citations about title and abstract, after full text, extracting data, and adjudicating risk of bias. Initially, two authors (IKS, FASSN) independently reviewed each article based on the title and abstract. Subsequently, the same authors independently evaluated the full text of the selected articles following the inclusion criteria. The final decision on the inclusion of studies was decided through consensus or by a third author (VSQ). We documented reasons for exclusion in full text only (supplementary material).

### Data extraction process

Two authors (IKS, FASSN) extracted the study characteristics, and a third author (PMSD) confirmed the data. When the studies had several publications with the same participants, we included the main study and extracted additional details from related references. When necessary, the authors of included studies were contacted via email for additional information, missing values, or data.

### Data synthesis and analysis

We evaluated heterogeneity between studies using the I^2^ statistic (<25%, low heterogeneity, 25–50%, moderate heterogeneity, and > 50%, high heterogeneity) [16]. If heterogeneity remained high, we excluded the analyses and reported the results of synthesis narrative. Data were reported as mean difference with 95% CI, and we used random-effects models. For studies with two or more groups of the comparator (another exercise type or control group) in the meta-analysis, we used data of the control groups only. It was not appropriate to conduct a quantitative analysis for the risk of publication bias, because there were fewer than ten studies (seven studies) included in the systematic review [17]. The meta-analysis was conducted using Review Manager (RevMan 5.4, Cochrane Collaboration) for data analysis and to generate figures following standard guidelines [18]. For primary and secondary outcomes, we assessed the certainty of evidence according to Grading of Recommendations Assessment, Development and Evaluation (GRADE) [19,20] using GRADE PRO software (https://gdt.gradepro.org). Two reviewers (IKS, RNC) evaluated the quality of evidence using GRADE and resolved discrepancies by consensus.

### Summary measurements

Primary outcomes were metabolic parameters [total cholesterol (TC), low-density lipoprotein (LDH-c), high-density lipoprotein (HDL-c), triglycerides (TG), fasting blood glucose, homeostatic model assessment (HOMA-IR), and fasting insulin]. The secondary outcomes of interest were body composition [weight (kg), body mass index (BMI), waist circumference (cm), body mass (%), body fat (%), and waist hip ratio (WHR)].

### Risk of bias (quality) assessment

We used the Tool for the Assessment of Study quality and reporting in Exercise (TESTEX) scale to evaluate the internal validity of the included studies [21]. A quality assessment tool and study report specifically for studies with physical training. This tool is based on 15 points (5 points for the quality of the study and 10 points for reports). Two authors (IKS, FASSN) independently assessed each study using excel and a third reviewer (RNC) resolved conflicts.

## 3. Results

**Fig 1:**
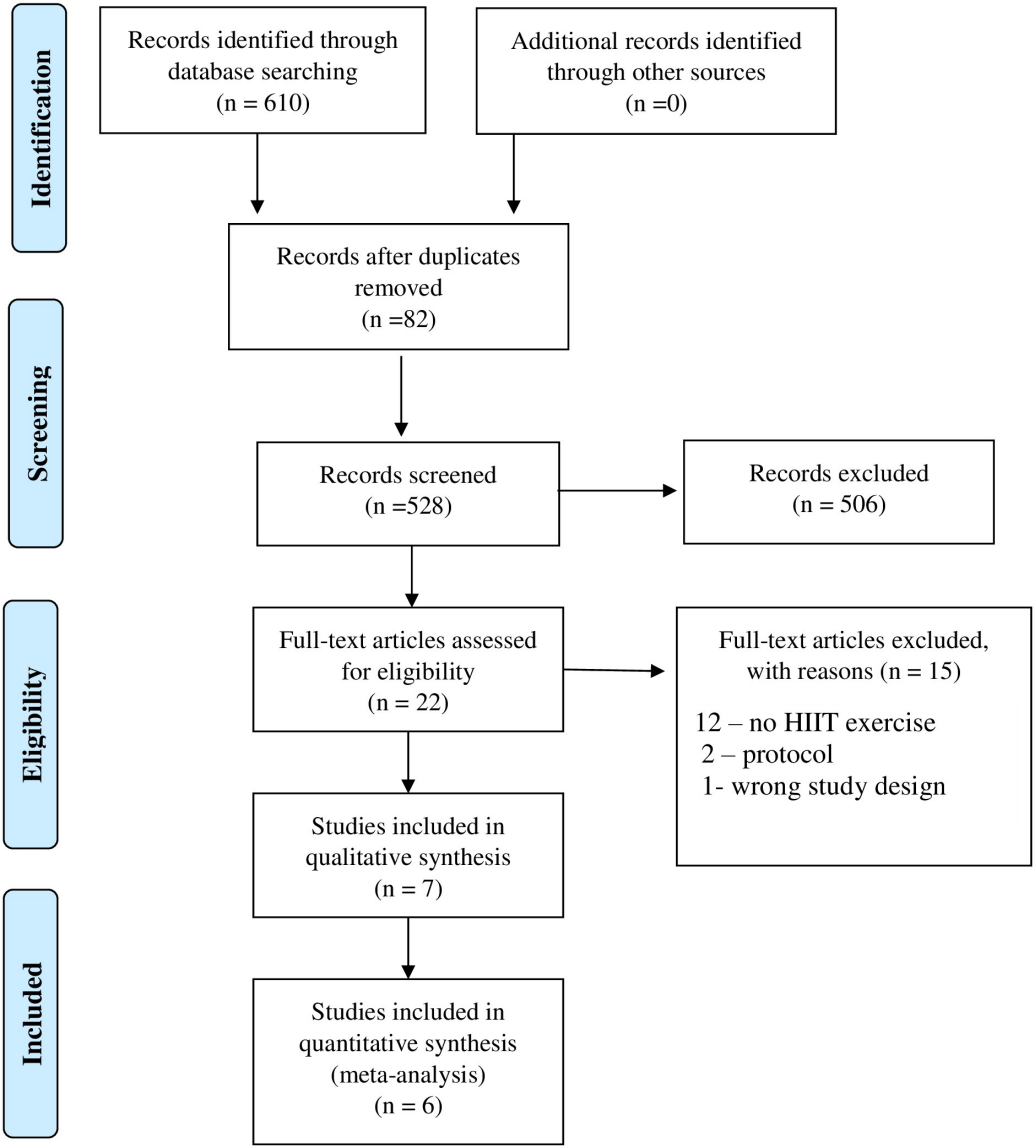
PRISMA flow diagram for the systematic review.

We identified 610 potentially eligible citations through electronic databases, and 82 duplicates were excluded. Of the 528 total citations, 506 were excluded at Level 1. At Level 2, 22 full-text publications were reviewed, and 15 were excluded (no study was included based on searches in references). In total, seven articles were included in the systematic review, and six articles were included in the meta-analysis (Fig 1).

### Characteristics of included studies

The included study characteristics are summarized in Table 2, and all data about excluded studies are supplementary material. Seven RCTs met the inclusion criteria (Almenning et al., 2015; Lopes et al., 2018; Aktaş et al., 2019; Faryadian et al., 2019; Ribeiro et al., 2019, 2020; Samadi et al., 2019) [22–28]. The studies were developed in different countries, such as Brazil, Iran, Turkey, and Norway. All studies reported the diagnosis of PCOS using the Rotterdam criteria. The number of participants in each trial ranged from 24 to 110 (total n = 423) participants. The mean age of the participants ranged from 18 to 40 years. Six RCTs included women that had a mean BMI classified as overweight or obese [22,23,25–28]; in only one study were the participants classified as “normal weight” [24].

**Table 2:** Characteristics of included studies (n = 7).

First Author Year	Study Location	Study Design	Duration of intervention	N	Participant Characteristics	Diagnosis of PCOS Criteria	Intervention	Comparators
Aktas et al., 2019 [22]	Turkey	CT quasi experimental	12 weeks	31	Age: 25.1 (4.6)	Rotterdam criteria	HIIT (walking/running)	MICT (running)
					BMI: 28.7 (6.9) kg/m^2^			
Almmening et al., 2015 [23]	Norway	RCT pilot	10 weeks	31	Age: 27.2 (5.5)	Rotterdam criteria	HIIT (walking/running and/or cycling)	Comparador 1: Strength training (eight dynamic strength drills with a resistance/machines)
					BMI: 26.7 (6.0) kg/m^2^			
								Comparador 2: Control group (recommended > 150 minutes of weekly)
Faryadiani et al., 2019 [24]	Iran	CT quasi experimental	12 weeks	24	Age: 34.34 (4.69)	Rotterdam criteria	HIIT) (running)	Control group (no intervention)
					BMI: 21.19 (1.74) kg/m^2^			
Lopes et al., 2018 [25]	Brazil	RCT	16 weeks	110	Age: 30.2 (5.1)	Rotterdam criteria	IAT (treadmills)	Comparador 1: CAT (treadmills)
					BMI: 29.9 (5.3) kg/m^2^			Comparador 2: Control group (no intervention)
Ribeiro et al., 2020 [26]^a^	Brazil	RCT	12 weeks	87	Age: 29.1 (5.3)	Rotterdam criteria	IAT (treadmills)	Comparador 1:CAT (treadmills)
					BMI: 29.1 (5.2) kg/m^2^			Comparador 2: Control group (no intervention)
Ribeiro et al., 2019 [27]	Brazil	RCT	16 weeks	110	Age: 29.1 (5.3)	Rotterdam criteria	IAT (treadmills)	Comparador 1: CAT (treadmills)
					BMI: 29.1 (5.2) kg/m^2^			Comparador 2: Control group (no intervention)
Samadi et al., 2020	Iran	RCT	12 weeks	30	Age: 20–35 years	Rotterdam criteria	Aquatic high intensity interval training (AHIIT)	Control group (use Metformin—1500 mg)
					BMI: 32.80 (4.49) kg/m^2^			

**Note** = MICT = Medium Intensity Continuous Training; CAT = continuous aerobic training; IAT = Intermittent aerobic training; HIIT = High Intensity Interval Training.

### Characteristics of interventions

**Fig 2:**
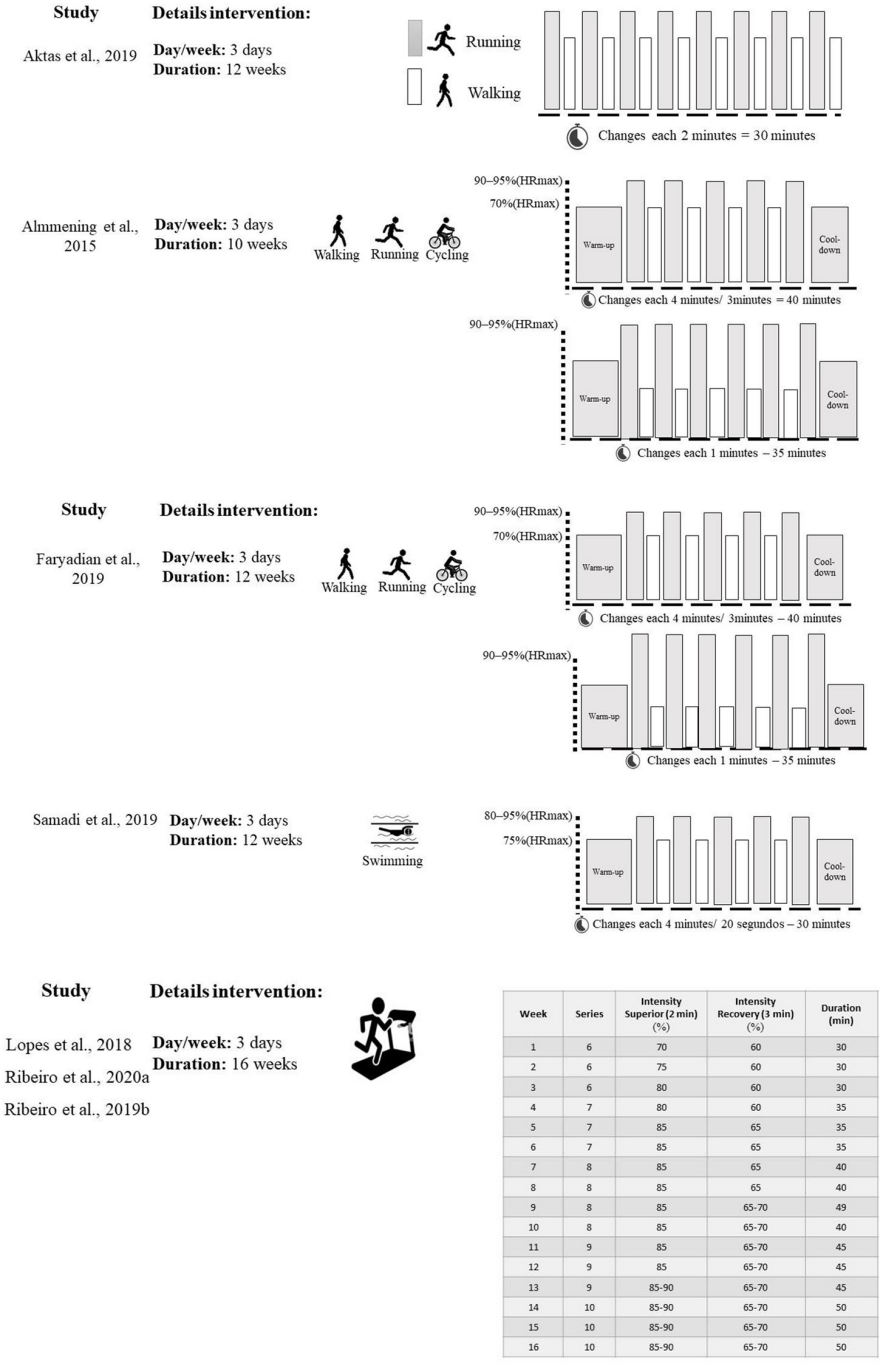


Only one study compared HIIT with a control group (without any intervention) [24] (Faryadian et al., 2019) [24]. One study compared aquatic high-intensity interval training (AHIIT) with a control group receiving 1500 mg of metformin [28]. Only one study compared high-intensity aerobic exercise vs. strength training with a control group (received recommended > 150 minutes per week) [23]. Four studies compared intermittent aerobic training vs. continuous aerobic training with a control group [22,25–27]. The duration of intervention for four studies was 12 weeks, 16 weeks for two studies, and 10 weeks for only one study. Details about the characteristics of the interventions included in the studies are described in Table 2. The intensity of exercise in most studies was moderate or vigorous, with values ranging from 60 to 95% of maximal heart rate or VO_2_ max. Participants in the included studies performed aerobic exercise (walking, running, and cycling) or aquatic exercise three times a week for 30–60 minutes per session. All details about HIIT intervention are described in Fig 2.

### Characteristics of the outcomes

#### Blood lipid profile

Five studies analyzed the effect of HIIT in comparison with moderate-intensity exercises or a control group on metabolic parameters, such as serum insulin levels, fasting glucose, TG, total cholesterol, LDL-c, HDL-c, and homeostatic insulin resistance (HOMA-IR). In four studies (Almenning et al., 2015; Aktaş et al., 2019; Faryadian et al., 2019; Samadi et al., 2019) [22,23,24,28], it was possible to observe that after HIIT, changes occurred in the homeostatic evaluation of insulin resistance (HOMA-IR). In addition, the results revealed a decrease in insulin resistance in women with PCOS. In two studies (Almenning et al., 2015; Aktaş et al., 2019) [22,23], the values of TG, total cholesterol, and LDL-C decreased, while HDL-C levels increased. In a study by Ribeiro et al. (2020) [26], the results revealed little evidence that HIIT could cause significant changes in these parameters.

#### Body mass and composition

Most of the included studies investigated the effect of HIIT on body composition (total arm mass, percentage of fat on the trunk, percentage of fat on the legs, and total gynoid mass) or anthropometric indices (body weight, BMI, WC, and WHR indexes). Only one study (Faryadian et al., 2019) [24] did not analyze the pre- and post-intervention anthropometric indices of women with PCOS. However, four studies (Lopes et al., 2018; Aktaş et al., 2019; Ribeiro et al., 2019, 2020) [22,25,26,27] observed a significant decrease in BMI and WHR values after HIIT intervention. In two studies (Almenning et al., 2015; Samadi et al., 2019) [23,28], the percentage of fat or fat mass decreased significantly in the group that performed HIIT.

#### Reproductive function (hormones)

Only four studies analyzed some hormonal parameters (follicle-stimulating hormone [FSH], luteinizing hormone [LH], sex hormone-binding globulin [SHBG], and testosterone [TST]) [23,25,26,28]. Only two studies reported information on the menstrual cycle (self-report) of the participants [23,28]. For example, Samadi et al. reported that, in both groups (HIIT and control), significant improvement was observed in setting menstrual cycles [28]. Only two studies reported data about LH and FSH [27,28]. Three studies reported data about TST [23,25,27], and there was also a significant effect in reducing the testosterone levels of women with PCOS after HIIT in two studies [25,27]. However, one study did not observe any significant changes in total serum testosterone after exercise training [23]. Four studies reported data about SHBG [23,25,27,28], and only two studies reported an increase after HIIT [25,28]. No meta-analysis was possible because of high clinical and statistical heterogeneity among studies [29].

### Risk of bias results

Table 3 reports the data of bias risk assessment for the included studies. According to the TESTEX scale (0–15 points), six studies had a score ≥ 9 points, and only one study had a score below 9 points. All studies reported the eligibility criteria, the baseline data of all participants clearly randomized, the statistical comparison between the groups, and the intensity related to the exercise remained constant. The weaknesses of the studies were related to the allocation of the group in a hidden way from patients eligible for inclusion in the study, evaluation of outcome measures in 85% of the participants, carrying out the intention-to-treat analysis (ITT), and monitoring activities in control groups.

**Table 3:** Assessment of study quality and reporting of included studies.

Authors, year	Study Quality																	Total
	1	2	3	4	5	Partial 0–5	6a	6b	6c	7	8a	8b	9	10	11	12	Partial 0–10	
Almmening et al.	1	1	0	1	0	3	1	1	1	NR	1	1	1	1	1	1	9	12
Aktas et al.	1	1	0	1	1	4	1	0	0	NR	1	1	1	0	1	0	5	9
Faryadian et al.	1	0	0	1	1	3	1	0	1	NR	1	1	1	0	1	0	6	9
Lopes et al.	1	1	1	1	1	5	0	1	1	NR	1	1	1	0	1	1	7	12
Ribeiro et al.	1	1	1	1	1	5	0	1	1	NR	1	1	1	0	1	1	7	12
Ribeiro et al.	1	1	1	1	1	5	0	1	1	NR	1	1	1	0	1	1	7	12
Samadi et al.	1	0	0	1	0	2	0	1	1	NR	1	1	1	0	0	0	5	7

NR—not reported; criteria: 1 –Eligibility criteria specified; 2 –Randomization specified; 3 –Allocation concealment; 4 –Groups similar at baseline; 5 –Blinding of assessor; 6 –Outcome measures assessed in 85% of participants; 7 –Intention-to-treat analysis 8 –Between-group statistical comparisons reported; 9 –Point measures and measures of variability for all reported outcome measures; 10 –Activity monitoring in control groups; 11 –Relative exercise intensity remained constant; 12 –Exercise volume and energy expenditure.

### Synthesis of results

We planned to synthesize all the results regarding the metabolic parameters and body composition of women with PCOS. However, the values of HDL-c (*I*^2^ = 96%) and fasting blood glucose (*I*^2^ = 95%) showed high heterogeneity between studies (even using random effects models), and it was not appropriate to combine data to calculate an overall effect [18]. We conducted subgroup analyses comparing different means of BMI (overweight and obesity) and comparing different exercise interventions (continuous aerobic training and strength training) with HIIT.

### 3.1 Meta-analysis

#### 3.1.1. Metabolic parameters (total cholesterol, LDL, insulin, and HOMA-IR)

**Fig 3:**
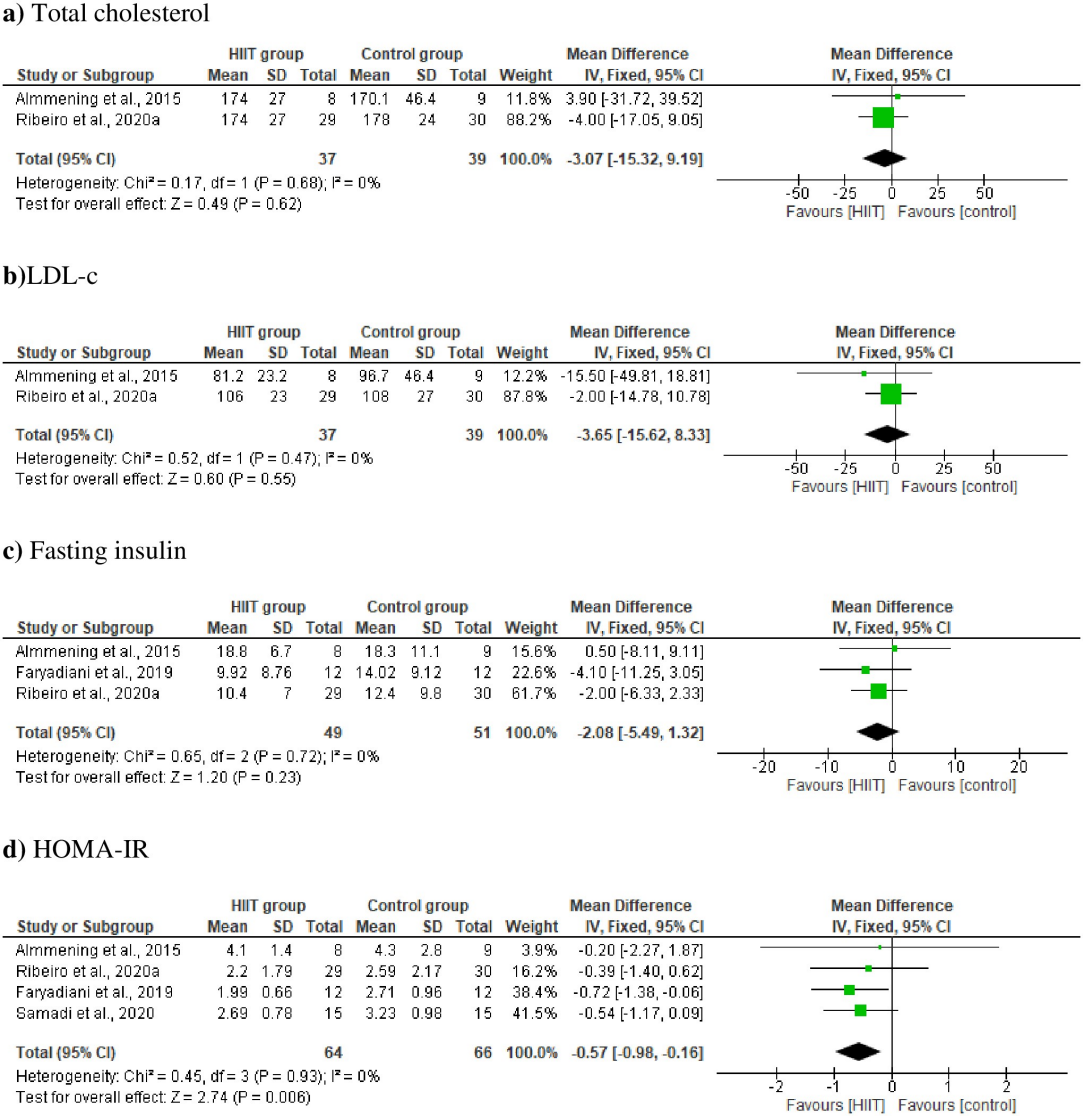
Change in metabolic parameters [a)Total cholesterol; b) LDL-c; c) fasting insulin and d) HOMA-IR] in HIIT group *vs* control group (no intervention).

Six studies were included in the meta-analysis that examined the effect of HIIT compared with the control group (no intervention received or another exercise type) on metabolic parameters from baseline to post-intervention. Comparing HIIT and control groups (no intervention) revealed no effect on total cholesterol (MD –3.07 mg/dL, [95% CI −15.32, 9.19], p = 0.62; Fig 3a), LDL-c (MD –3.65 mg/dL, [95% CI −15.62, 8.33], p = 0.55; Fig 3b), and fasting insulin (MD –2.08 mg/dL, [95% CI −5.49, 1.32], p = 0.23; Fig 3c) in women with PCOS. There was no significant heterogeneity across studies. The summary coding revealed that HIIT significantly decreased HOMA-IR (MD– 0.57, [95% CI −0.98, -0.16], p = 0.0006; Fig 3d) in women with PCOS, with no significant heterogeneity across studies (*I*^2^ = 0%; p = 0.93). Details of the effect estimate and GRADE certainty ratings are summarized in S1 Table.

#### 3.1.2. Body composition

**Fig 4:**
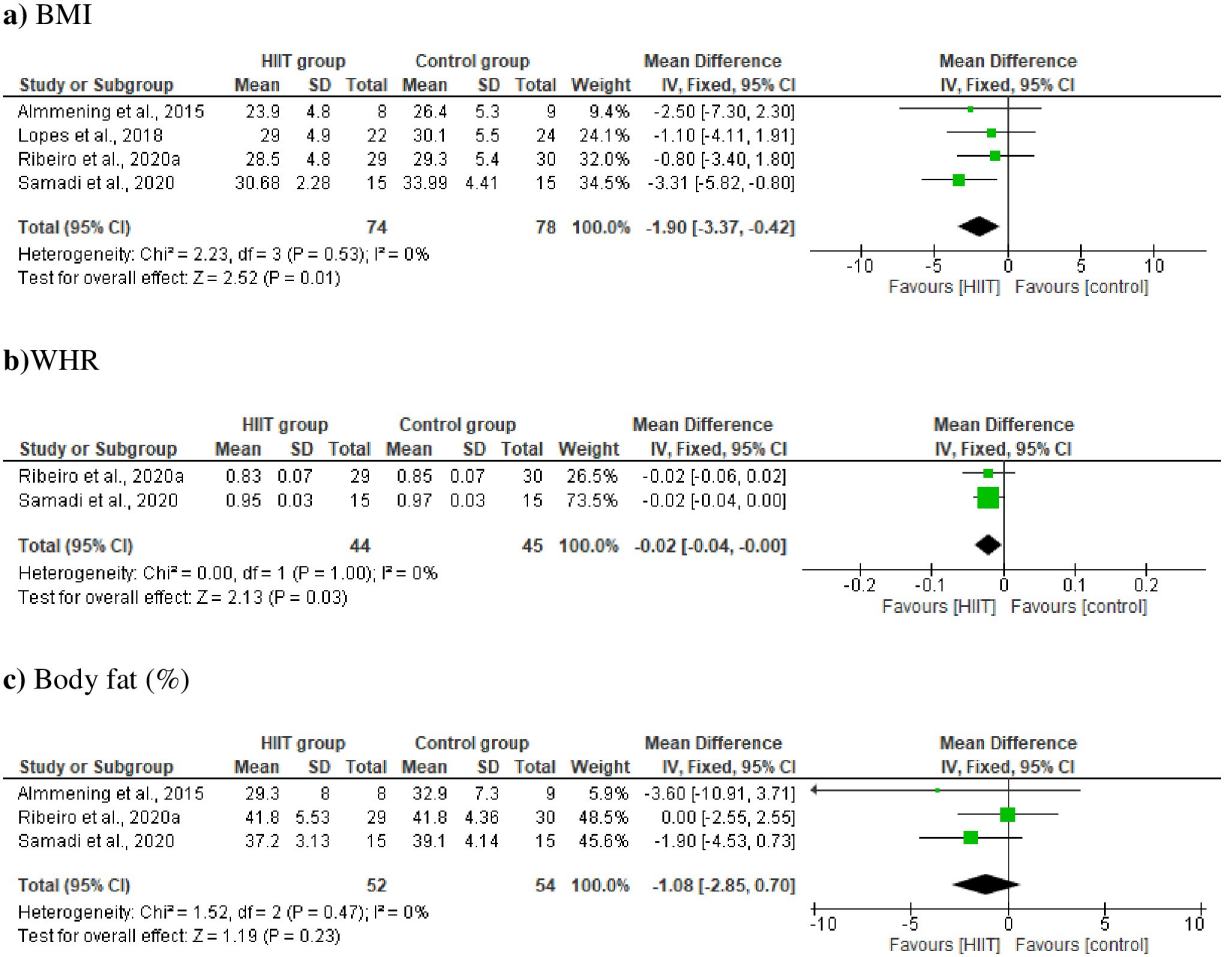
Change in body composition [a) BMI; b) WHR and c) body fat (%)] in HIIT group vs control group.

Five studies with HIIT vs control group (no intervention) showed significant effect on BMI (MD –1.90 kg/m2, [95% CI −3.37, −0.42], p = 0.01; Fig 4a) in women with PCOS. There was a high certainty of evidence of lower BMI favoring the HIIT intervention. The summary coding of two studies revealed significant effect on WHR (MD –0.02, [95% CI −0.04, 0.00], p = 0.03, Fig 4b). Comparisons of HIIT and control groups (no intervention) revealed no significant differences in body fat (%) (MD −1.08%, [95% CI −2.85, 0.70], p = 0.23, Fig 4c). There was no significant heterogeneity across studies (Fig 4).

### 3.2. Subgroup (BMI <29.0 kg/m^2^ >29.9 kg/m^2^)

**Fig 5:**
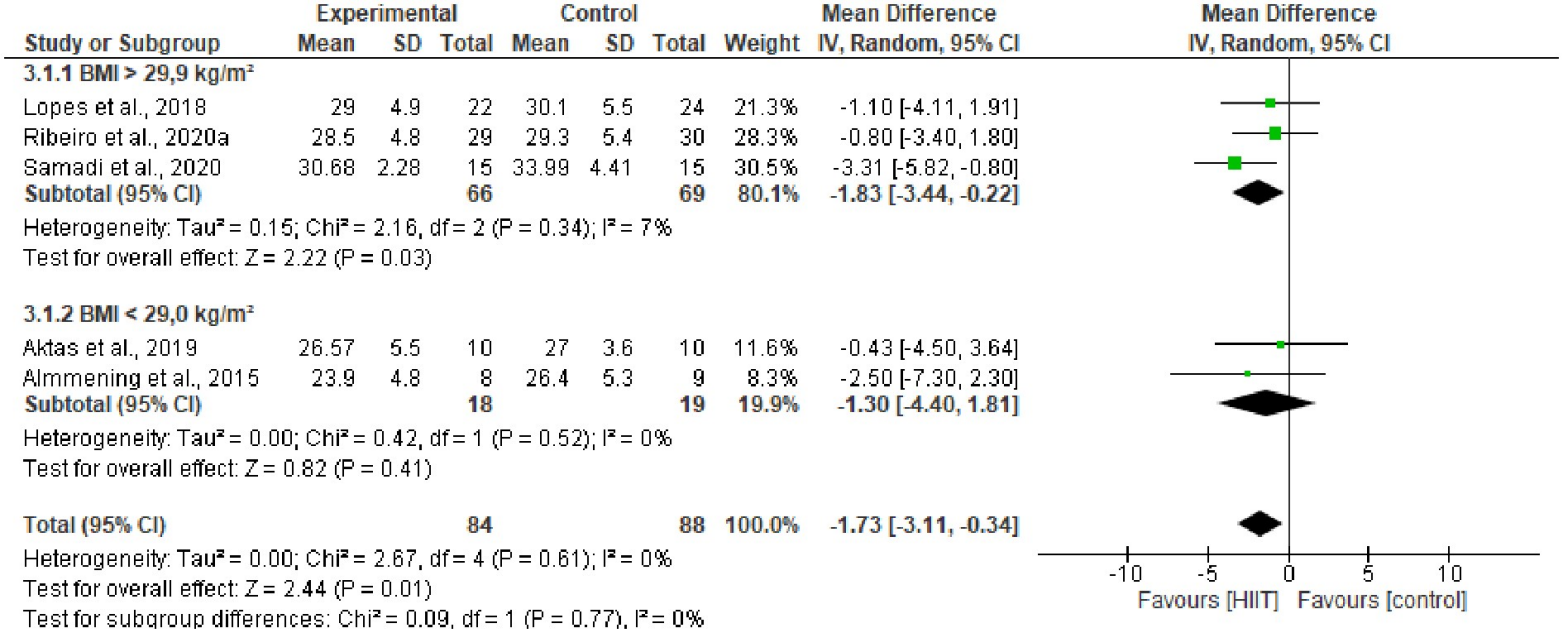
Change in body composition subgroup [BMI] in HIIT group vs control group.

Subgroup analyses were performed to compare the effects of HIIT on the BMI of overweight and obese women. The results showed that HIIT training favored the loss of total absolute fat mass (kg) (-1.82 [95% CI -3.44 to -0.22], p = 0.03) (Fig 5).

#### 3.2.1. Subgroup (HIIT vs. other exercise intervention)

**Fig 6:**
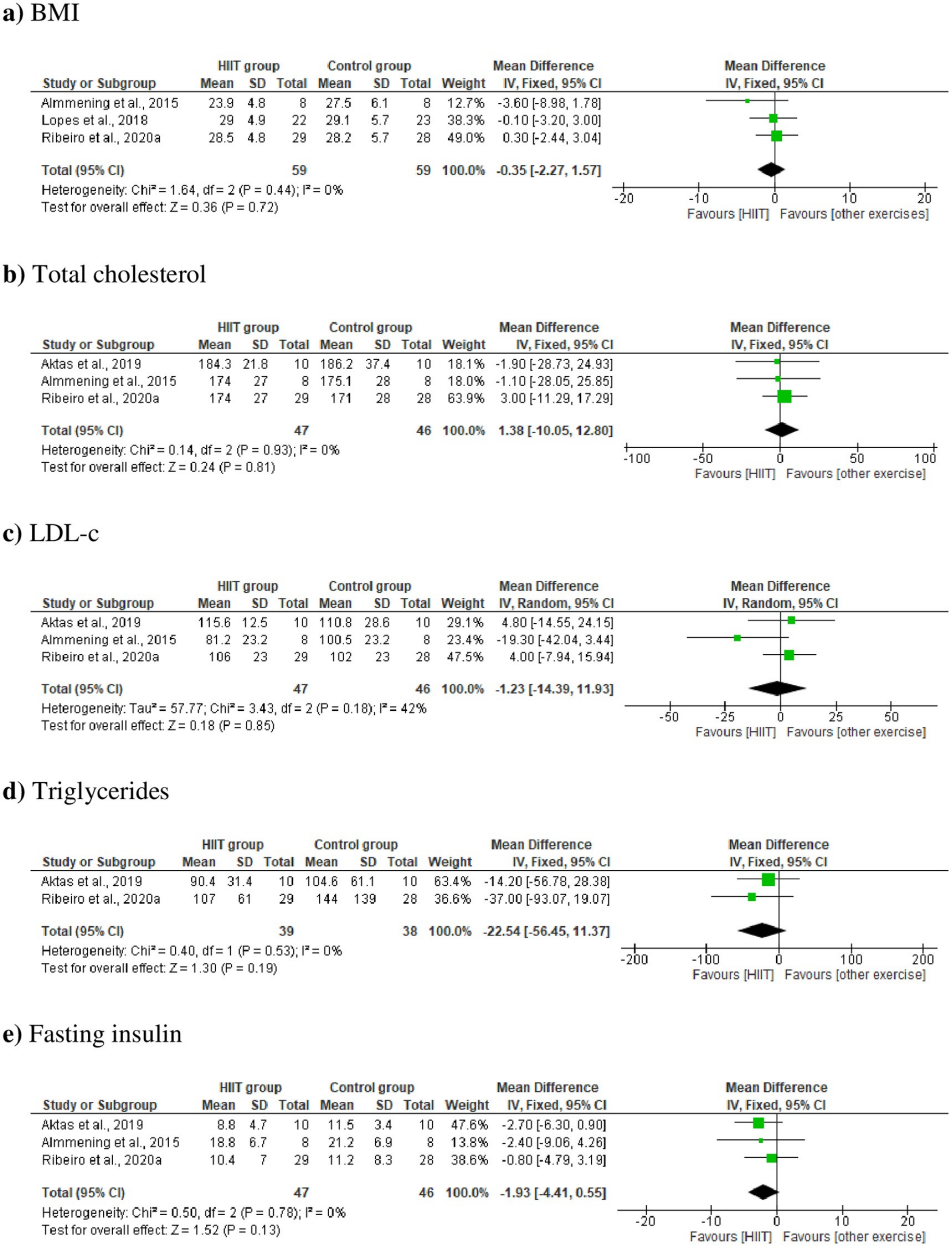
Change in subgroup a) BMI, b) total cholesterol, c) LDL-c, d) triglycerides, and e) fasting insulin in HIIT vs. other exercises.

Subgroup analyses were performed to compare the effects of HIIT and other intervention (continuous aerobic training or strength training) on the BMI, total cholesterol, LDL-c, triglycerides, and fasting insulin in women with PCOS. The results showed no significative effect on BMI (MD −0.35 kg/m2 [95% CI −2.27, 1.57], p = 0.72; Fig 6a), total cholesterol (MD 1.38 mg/dL, [95% CI −10.50, 12.18], p = 0.81; Fig 6b), LDL-c (MD –1.23 mg/dL, [95% CI −14.39, 11.93], p = 0.85; Fig 6c), triglycerides (MD −22.54 mg/dL, [95% CI −56.45, 11.37], p = 0.19; Fig 6d), and fasting insulin (MD –1.93 mg/dL, [95% CI −5.49, 1.32], p = 0.23; Fig 6e) (Fig 6).

### 3.3. Sensitivity analysis

Sensitivity analyses were carried out to explain possible heterogeneity among studies by including studies in, or excluding them from, the meta-analysis based on the study design and sample. Two studies were excluded from the analysis of WHR and triglycerides [23,25]. After the sensitivity analysis, a significant difference was observed between the groups and a low heterogeneity in the WHR analysis (I^2^ = 0%; p = 0.03; Fig 4b), different from the previous analysis (I^2^ = 22%, p = 0.74). On the other hand, in the analysis of triglycerides, a low heterogeneity was observed (I^2^ = 0%; p = 0.19; Fig 6d); however, it remained without significant difference and did not change the results of the first meta-analysis.

## 4. Discussion

HIIT was more efficient than other interventions (moderate exercise or no intervention) for reducing HOMA-IR and BMI in women with PCOS with moderate and high certainty of evidence. However, there was no significant reduction in other outcomes assessed. Based on the results of the potential of HIIT to reduce HOMA-IR parameter in women with PCOS, the possible mechanisms of this action may be linked to muscle contraction from physical exercise, resulting in the translocation of GLUT-4 receptors inside the cell of the membrane, facilitating the diffusion of plasma glucose into striated muscle tissue and adipocytes without the need for insulin action. Thus, when performing regular exercises, metabolic and hormonal adaptations contribute to a significant increase in insulin receptors [29,30].

In addition, the potential of HIIT to reduce the HOMA-IR parameter in women with PCOS may be related because it involves several minutes of high-intensity exercise (for example, ≥90% VO2 max or maximum heart rate). HIIT is a time-efficient training approach to stimulate biogenesis and increase skeletal muscle insulin sensitivity due to improvement of the mitochondrial function of the muscle; that is, the activation of the mitochondria receptors allows more energy to be produced and improves the maximum level of activity, increasing the skeletal oxidation capacity. In addition, antioxidant adaptations are superior in HIIT programs compared to continuous exercise of moderate intensity [31]. In part, these findings could justify a superior effect of HIIT in reducing the HOMA-IR parameter in relation to moderate-intensity exercise considering that oxidative stress seems to play an important role in the pathogenesis of insulin resistance [32,33].

Studies using animal models have found that antioxidant adaptations are superior in HIIT programs compared to continuous exercise of moderate intensity [34,35]. Little et al. observed that, after HIIT sessions, the practical model is a potent stimulus to increase the mitochondrial capacity of the human skeletal muscle and is effective in improving muscle metabolic capacity and functional performance [36]. Racil et al. reported that HIIT positively changes blood lipids and adiponectin variables in obese adolescent girls, resulting in improved insulin sensitivity, as indicated by a lower HOMA-IR [37].

There is substantial evidence to support the effectiveness of exercise in improving glucose homeostasis. A meta-analysis investigating the effects of HIIT on glucose regulation markers and insulin resistance in adults identified a reduction in HOMA-IR after HIIT, suggesting that HIIT may improve insulin sensitivity in those who are resistant. In this sense, HIIT can be an efficient treatment strategy for women who at a high risk of developing diabetes mellitus [38]. Thus, this reduction in HOMA-IR in the studied population may be efficient for treating the syndrome and its possible consequences, since women with PCOS have a higher prevalence of insulin resistance (IR), hyperinsulinemia, dyslipidemia, and metabolic syndrome [39].

Furthermore, the results of the meta-analysis showed that HIIT reduced the BMI of women with PCOS, supporting evidence that high-intensity exercise causes higher consumption of oxygen and greater post-exercise energy balance, leading to favorable results in the individuals’ body composition. For women with PCOS, the reduction in body mass can help control some consequences of PCOS, such as improvement in menstrual and reproductive function, in addition to influencing psychosocial aspects [40].

Interestingly, after performing the subgroup analyses, it was possible to verify that the effect of HIIT on BMI was more pronounced in women with PCOS who were obese. It is possible for individuals with a higher BMI to obtain greater benefits after engaging in HIIT programs, at least regarding changes in body composition. Research conducted found that young people with BMI above 25 kg/m^2^ significantly reduced body mass (BM) and percentage of body fat after eight weeks of HIIT (~ 3x / week), while young people with BMI less than 25 kg/m^2^ did not show significant differences in these variables [41]. Similarly, a study identified that adolescents with BMI above 25 kg/m^2^ showed greater reductions in the percentage of body fat, WHR, BM, and BMI after ten weeks of HIIT (~ 1x / week), in relation to their pairs with BMI below 25 kg/m^2^ [42]. A single study included in our review looked at women with normal BMI (BMI <25 kg/m^2^) [24]. Therefore, it was not possible to verify the impact of HIIT on this outcome in this group.

Previously, excess adiposity was found to be associated with worsening health in women with PCOS, and changes in body composition due to lifestyle changes (~ 6 months) were associated with the return of ovulation in obese women with PCOS [43,44]. This evidence provides evidence that the effects of physical exercise on the health of women with PCOS may be linked to reduced adiposity. However, this theory may not apply to women with PCOS who have a normal BMI. We speculate that adiposity in this population does not significantly reduce as a result of physical exercise and, therefore, other factors are important for improving health caused by this type of intervention [24]. The scarcity of studies dealing with women with eutrophic PCOS limits the understanding of the role of physical exercise in the health of this population. Possibly, most authors choose to select overweight/obese groups due to the high prevalence of PCOS [45].

This systematic assessment of HIIT interventions supports interesting results on the effectiveness of HIIT and aerobic training on this population. However, the difference between the duration of the intervention (i.e., 10, 12, and 16 weeks) and number of participants (n = 24 to 110) in each RCT may have influenced some results. The lack of positive results in the percentage of fat and waist circumference after the analyses performed may have suffered this influence. Moreover, they can be explained by the need for aid of a dietary intervention to promote future improvements. Although the evidence is clear about the benefits of planned exercises for the treatment of PCOS, studies indicate that combining exercises with planned diet (i.e., lifestyle) and psychological strategies can be an important strategy for the management of PCOS [46].

This study has some limitations, such as heterogeneity between studies due to the variety in the duration of interventions and the small number of participants in the studies. In addition, the methodological quality analyzed through TESTEX showed that the studies had limitations due to the lack of allocation of groups in a hidden way, blindness of the evaluator, lack of carrying out analyses for the purpose of treating (ITT), and monitoring activities in groups of patients control. Despite some limitations, we were able to explore and synthesize the effects of high-intensity training doses on the worrying outcomes experienced by women with PCOS.

This large set of data may assist in future exercise prescriptions for this population. To the best of our knowledge, this is the first systematic review with meta-analysis on the effects of HIIT on results related to metabolic health, in which the results help expand the discussions and conclusions of other studies. Finally, we suggest that the weaknesses observed in these studies can be included in future studies.

## 5. Conclusion

HIIT alone is an effective strategy for reducing HOMA-IR and BMI in women with PCOS. The evidence is limited to discern the effect of HIIT on the other results obtained; however, the analyses showed that high-intensity training improved some clinical outcomes in this population, supporting the role of exercise as a non-pharmacological treatment for women with PCOS. This review may provide some evidence and suggestions for the clinical application of HIIT in PCOS. Future studies should be conducted with a longer duration (> 16 weeks), larger sample sizes, and more detailed results.

## Supporting information

S1 Checklist PRISMA checklist 2009. (PDF)

S1 Table Summary of findings of clinical trials comparing the effects of HIIT on metabolic parameters and body composition of women with polycystic ovary syndrome after 10 to 16 weeks. (DOCX)

S1 File Characteristics of excluded studies. (DOCX)
