# Supplementary material for: Effect of high-intensity interval training on metabolic parameters in women with polycystic ovary syndrome: A systematic review and meta-analysis of randomized controlled trials
Source: PLoS One. 2021 Jan 19;16(1):e0245023. doi: 10.1371/journal.pone.0245023 (PMC7815156; doi:10.1371/journal.pone.0245023)
Supplement: S1 Table — (DOCX) [file pone.0245023.s002.docx]

**Table 4.** Summary of findings of clinical trials comparing the effects of HIIT on metabolic parameters and body composition of women with polycystic ovary syndrome after 10 to 16 weeks.

| Outcomes* | № of participants  (studies)  Follow up | Absolute  (95% CI) | Certainty of the evidence (GRADE)^2^ |
| --- | --- | --- | --- |
| Total Cholesterol | 96 (3 RCTs) | MD **2.89 higher** (14.01 higher to 8.28 higher) | ⨁⨁◯◯ LOW |
| low-density lipoprotein  (LDL-c) | 96 (3 RCTs) | MD **1.3 lower**  (11.49 lower to 8.88 higher) | ⨁⨁◯◯ LOW |
| Fasting insulin | 120 (4 RCTs) | MD **2.38 lower** (4.85 lower to 0.1 higher) | ⨁⨁⨁◯ MODERATE |
| HOMA-IR | 130 (4 RCTs) | MD **0.57 lower** (0.98 lower to 0.16 lower) | ⨁⨁⨁◯ MODERATE |
| Triglycerides | 96 (3 RCTs) | MD **7.08 higher** (17.9 lower to 32.07 higher) | ⨁⨁◯◯ LOW |
| Body mass index (BMI) | 172 (5 RCTs) | MD **1.73 lower** (3.11 lower to 0.34 lower) | ⨁⨁⨁⨁ HIGH |
| Body fat | 106 (3 RCTs) | MD **1.08 lower** (2.85 lower to 0.7 higher) | ⨁⨁⨁⨁ HIGH |
| Waist Hip Ratio (WHR) | 135 (3 RCTs) | MD **0.01 lower** (0.04 lower to 0.03 higher) | ⨁⨁⨁◯ MODERATE |

Note: * Primary outcome measure: the outcome is a change from baseline; CI: Confidence Interval; RCTs: randomized controlled trial; GRADE: Grading of Recommendations Assessment, Development and Evaluation;

1 Method of analysis for all outcomes: random effect

2 About the certainty of the evidence (GRADE): High certainty: very good indication of the likely effect (confident that the true effect lies close to that of the estimate of the effect); Moderate certainty: good indication of the likely effect (the true effect is likely to be close to the estimate of the effect, but there is a possibility that it is substantially different); Low-certainty: the confidence in the effect estimate is limited (the likelihood that it will be substantially different); Very Low-certainty: have very little confidence in the effect estimate.
