# Supplementary material for: Effect of high-intensity interval training on metabolic parameters in women with polycystic ovary syndrome: A systematic review and meta-analysis of randomized controlled trials
Source: PLoS One. 2021 Jan 19;16(1):e0245023. doi: 10.1371/journal.pone.0245023 (PMC7815156; doi:10.1371/journal.pone.0245023)
Supplement: S1 File — (DOCX) [file pone.0245023.s003.docx]

| **Authors, years** | **Reason for exclusion** |
| --- | --- |
| Hiam et al., 2019 | Wrong design (protocol) |
| Roessler et al., 2013 | Wrong intervention (cross over) |
| Bruner, Chad, Chizen 2006 | Wrong intervention da (Resistance exercise) |
| Sprung et al., 2012 | Wrong intervention (no HIIT) |
| Kazemi et al., 2020 | Wrong intervention (no HIIT) |
| McBreairty et al., 2020 | Wrong intervention (no HIIT) |
| Tiwari et al., 2019 | Wrong intervention (no HIIT) |
| Cutler et al., 2018 | Wrong design (protocol) |
| Kazemi et al., 2018 | Wrong intervention (no HIIT) |
| Costa et al., 2018 | Wrong intervention (no HIIT) |
| Sá et al., 2016 | Wrong intervention (no HIIT) |
| Nybacka et al., 2011 | Wrong intervention (no HIIT) |
| Vigoritto et al., 2007 | Wrong intervention (no HIIT) |
| Thomson et al., 2010 | Wrong intervention (no HIIT) |
| Hutchison et al., 2011 | Wrong design (no RCT) |
| Zademodares et al., 2018 | Wrong design (no RCT) |

(Supplementary material)

**Characteristics of excluded studies**
